# Shelf Life Prediction of Longan with Intermediate Moisture Using Osmotic Dehydration, Combined with Different Packaging and Storage Temperatures

**DOI:** 10.3390/foods15010040

**Published:** 2025-12-23

**Authors:** Hong Phuc Vu Le, Napapan Chokumnoyporn, Jurmkwan Sangsuwan, Witoon Prinyawiwatkul, Sujinda Sriwattana

**Affiliations:** 1 Faculty of Agro–Industry, Chiang Mai University, Chiang Mai 50100, Thailand; hongphuc900511@gmail.com (H.P.V.L.); jurmkwan.s@cmu.ac.th (J.S.); 2 Culinary Art and Kitchen Management Division, Faculty of Science, Chandrakasem Rajabhat University, Bangkok 10900, Thailand; pnapapan@hotmail.com; 3 School of Nutrition and Food Sciences, Louisiana State University, Agricultural Center, Baton Rouge, LA 70803, USA; wprinya@lsu.edu

**Keywords:** fruit preservation, consumer testing, shelf–life prediction, aluminum foil laminated with plastic bag, health and the environment, small-scale farmer productivity

## Abstract

This study aimed to evaluate the shelf life of intermediate moisture longan (IML). A hurdle technology approach was applied, combining osmotic dehydration (OD), hot-air drying, and packaging methods: aluminum foil-laminated plastic bags with nitrogen flushing (Al bag with nitrogen), aluminum foil-laminated plastic bags without nitrogen (the Al bag without nitrogen), and clear plastic bags. Samples were stored at 4, 25, 35, and 45 °C for 24 weeks (six months). The combination of these preservation techniques was effective in extending the shelf life of IML products. Quality changes in IML during storage were significantly influenced by packaging type, storage temperature, and storage duration (*p* ≤ 0.05). Products stored in all three types of packaging at low temperatures retained better color (L* 31.92 ± 0.97–32.67 ± 1.47) and higher sensory scores (6.5 ± 1.4–6.6 ± 1.5) compared to those stored at higher temperatures (L* 19.54 ± 1.00–20.90 ± 1.48, 3.3 ± 1.6–4.1 ± 1.7). Accelerated shelf life testing using the Arrhenius equation was applied to predict changes in color and sensory acceptance. The kinetics of these quality changes followed the first-order reaction models. Among the packaging types, IML stored in Al bags with nitrogen exhibited the lowest rate constants, indicating slower quality deterioration and better protection compared to Al bags without nitrogen and clear plastic bags. The predictive model demonstrated strong agreement with the experimental data, accurately predicting shelf life at 25 °C and above. However, the model projected a potential shelf life of up to 58 weeks for IML samples packaged in aluminum bags with nitrogen and stored at 4 °C; this projection extended beyond the 24-week experimental period, which still verified a minimum shelf life of 24 weeks. This technology reduces post-harvest food loss, advances packaging innovation for agro-industry, and strengthens food security.

## 1. Introduction

Extending the shelf life of fruits and vegetables while maintaining their quality is a major challenge in food preservation. Several methods have been applied to slow enzymatic browning and microbial decay, which are the main causes of deterioration. Among them, drying or dehydration remains one of the oldest and most effective preservation techniques [1]. In particular, osmotic dehydration (OD) has gained attention because it reduces moisture content and water activity to the range of 0.6–0.84 by immersing products in aqueous solutions containing one or more solutes [2,3,4,5]. Intermediate moisture foods (a_w_ ≈ 0.6) are meant to be soft, chewy, and palatable without refrigeration. At this a_w_, microbial spoilage is prevented and good texture is maintained (not too hard, like fully dried fruit). So, 0.6 is a balance between safety, stability, and sensory quality. This process not only partially removes water but also improves stability against microbial growth and enzymatic activity. The addition of citric acid to osmotic solutions has been successfully used to inhibit enzymatic browning in longan fruit [6]. Moreover, when combined with hot-air drying, OD can significantly reduce drying time, lower energy consumption, and improve the quality of fruits and vegetables during storage [7,8,9,10].

Shelf life is generally defined as the period between food production and packaging and the point at which the product becomes unacceptable under given storage conditions [11]. To predict and evaluate shelf life, chemical kinetic models are widely used to describe quality changes during storage. The Arrhenius relationship has been applied to explain the effect of temperature on reaction rate constants [12] and to determine activation energy values for food quality losses [13]. Such approaches have been applied to modeling color and pH changes in bulk tomato paste containing olive leaf extract, color change in dried pomegranate arils during microwave–vacuum drying, and quality attributes of dried longan obtained through osmotic dehydration [14,15,16].

Alongside processing methods, packaging also plays a crucial role in extending shelf life. Modified atmosphere packaging (MAP), vacuum packaging, and shrink packaging are among the most common approaches [17]. Carbon dioxide, oxygen, and nitrogen are frequently used gases in MAP [18]. For example, carica seed powder stored in aluminum foil-laminated bags had a predicted shelf life of over one year under room temperature accelerated storage conditions [19]. Similarly, vacuum-dried coconut milk powder packed in aluminum foil-laminated polyethylene had a predicted shelf life of 30 days at 38 ± 2 °C [20]. For dried pomegranate arils, aluminum foil-laminated bags provided better protection compared with high-density polyethylene during accelerated storage [21]. These studies highlight the importance of selecting suitable packaging materials to maximize shelf life.

Longan (*Dimocarpus longan* L.), a fruit belonging to the family *Sapindaceae*, is a significant fruit crop, particularly in Southeast Asia. Thailand and Vietnam are the world’s leading producers and exporters. The longan production in eight northern provinces of Thailand in the year 2025 is approximately 1,064,242 tons. The Ministry of Commerce plans to export 15,000 tons of fresh longan and produce 50,000 tons of dried longan. Longan is highly perishable, with its short storage life mainly limited by pericarp browning and microbial decay [22,23,24]. The fungal pathogen *Lasiodiplodia theobromae* is reported as the most severe cause of decay in longan fruit [25]. Under normal conditions, longans can be stored for only a few days at 30 °C or up to 30 days at 4–5 °C after harvest [26]. Drying or other processing techniques can prolong the fruits’ storage life. Dried longan is a highly valued product commonly used in snacks, desserts, and traditional Chinese medicine. The main deterioration of dried longan is primarily moisture re-absorption and subsequent microbial spoilage, especially by yeast and mold, followed by quality degradation by, for instance, browning. Intermediate moisture longan requires precise partial drying or osmotic dehydration with preservatives and moisture-proof packaging, while fully dried longan involves a longer, higher-temperature drying to achieve very low moisture levels and robust packaging to prevent quality loss. Intermediate moisture longan is soft, pliable, and chewy, like fresh raisin, allowing it to be eaten as a ready-to-eat snack. Fully dried longan is hard, brittle, and leathery, sometimes requiring rehydration before consumption, as it has the texture of a hard, dried chip. Therefore, integrating osmotic dehydration with appropriate packaging and storage conditions could provide an effective solution to extend the shelf life of intermediate moisture longans (IMLs), preserving product quality and sensory attributes, facilitating market expansion, and reducing economic losses and food waste.

The prediction of shelf life for intermediate moisture foods (IMFs) has traditionally relied on water activity (a_w_) as the primary determinant of microbial stability [10]. Consequently, most existing models assume that shelf life is limited by microbial spoilage. However, a critical research gap exists for IMF products in which chemical and physical degradations, rather than microbial growth, become the dominant shelf life-limiting factors [27]. Specifically for intermediate moisture longan (IML), our preliminary observations indicated that a_w_ was not the crucial determinant of shelf life; instead, consumer rejection was primarily driven by color deterioration associated with non-enzymatic browning. Some previous studies validated kinetic models linking a browning rate to sensory acceptability, but these approaches have rarely been applied to IMF systems. Therefore, the current study addressed this gap by moving beyond the a_w_-centric perspective to develop a sensory-driven shelf life prediction model. The kinetic parameters of browning serve as a critical quality index directly correlated with consumer acceptance, providing a practical and mechanistic basis for predicting the shelf life of IML according to its true failure mode [27].

The objective of this study was to evaluate the shelf life of IMLs produced by osmotic dehydration and subsequently stored in different packaging types at different storage temperatures for six months.

## 2. Materials and Methods

### 2.1. Intermediate Moisture Longan Preparation

The longan fruits were purchased from Thongpoon Food Limited Partnership (Lamphun, Thailand) and delivered to faculty of Agro–Industry, Chiang Mai University within one day. After arriving, the fruits were cleaned, deseeded, and peeled. According to Layeghinia et al. [28], osmotic solutions for fruits and vegetables may include salt, sugar, sorbitol, and glycerol. In this study, the peeled longan fruits were then immersed in the osmotic solution [29], containing sugar solution (40% sucrose/glycerol/sorbitol solution at the ratio of 2:1:1, constituting 40% (*w*/*v*) of the total solution), potassium sorbate (0.1%), and citric acid (1%). In the preparation of the osmotic solution, 5% of the total water was used to dissolve potassium sorbate and citric acid. Subsequently, sucrose was dissolved in the remaining water according to the formulation, followed by the addition of glycerol and sorbitol. Then, the dissolved citric acid and potassium sorbate were added in the mixture solution and stirred until homogeneous. The fruit-to-solution ratio was 1:2. The treated longan was immersed for 12 h in the air-conditioned room (24 °C) without agitation. The fruits were then taken out of the osmotic solution, drained for 15 min and dried in a hot air oven (Binder oven FD 115, Tuttlingen, Germany) at 60 °C with an air velocity of 0.5 m/s for 8 h to bring the water activity down to 0.6. Then, the intermediate moisture longan (IML) products were kept in three different types of packaging: aluminum foil-laminated plastic bag flushed with nitrogen (Al bag with nitrogen), aluminum foil-laminated plastic bag flushed without nitrogen or air pack (Al bag without nitrogen), and clear plastic bag (polyamide) with an air pack. In order to accelerate physicochemical reactions, IML samples were stored at different temperatures (4, 25, 35, and 45 °C) and the quality of IML samples from different treatments were evaluated for physicochemical properties, microbial quality, and sensory acceptance at various time intervals. All packages were visually inspected for seal integrity after heat sealing, and the ones with visible defects were discarded.

### 2.2. Physicochemical Properties

Water activity (a_w_) was analyzed in triplicate using a Water activity analyzer (Decagon, Pullman, WA, USA). Two grams of the sample were placed in a water activity sample cup. The measurement was conducted in a controlled-temperature room at 23 °C. Water activity sample cups were equilibrated by placing the samples in the 23 °C room. Moisture content (MC) was analyzed in triplicate by a vacuum oven at 70 °C [30]. A colorimeter (Konica Minolta Chroma Meter, CR-410, Tokyo, Japan) was used to measure the color parameters of the 10 samples from each treatment and expressed as L*, a*, b*. The colorimeter was standardized with a white calibration plate (L* = 85.4, a* = 0.3176, and b* = 0.3341). The following colorimeter settings were used: a mode of D65 and a 0 °C viewing.

The hardness was evaluated by a penetration test using a Texture Analyzer model TA–XT2i (Texture Technologies, Inc., Godalming, UK). The penetration probe of 2 mm in diameter was applied. The pre-test speed was set at 1 mm/s, with a distance of 15 mm/s and an acquisition rate of 400 points/s. The measurement was determined in 10 replications for each sample.

### 2.3. Microbial Analysis

Total bacterial count (TBC), yeast and mold count (YMC), and *Escherichia coli* count at day 0 were determined as described by AOAC [31] to assess food safety, measure the effectiveness of the osmotic process in reducing microbial load, and monitor for spoilage-causing organisms. These tests help ensure that the product is safe to eat, has an acceptable shelf life, and meets regulatory standards by identifying the presence of pathogenic bacteria like *E. coli* and spoilage microorganisms like yeasts and molds, which can grow even under high-sugar conditions. Individual IML samples (10 g) were aseptically homogenized with 90 mL of peptone water (0.1%) using a stomacher for 1 min. Serial dilutions were made in 9 mL peptone water (0.1%) and pour plate was performed in duplicate. The media employed were Plate Count Agar (PCA), Potato Dextrose Agar (PDA), Lactose Broth (LSB) and Brilliant Green Lactose Bile Broth (BLGBB) for determination of TBC, YMC, and *E. coli*, respectively. The PCA plates were incubated at 35 °C for 48 h, the PDA plates were incubated at 25 °C for 72 h, and the LSB and BGLB tubes were incubated at 35 °C for 24–48 h [32]. The growth colonies were counted at the end of the incubation time and expressed as colony forming units per gram (cfu/g) of sample. The microbial analyses of IML samples were undertaken periodically during storage.

### 2.4. Consumer Acceptance

The consumer acceptance test was carried out every 3 weeks, according to the standard procedure [33], (N = 50 consumers) using a 9–point hedonic scale (1= “dislike extremely”, 5 = “neither like nor dislike”, 9 = “like extremely”). The sensory attributes considered were overall acceptability, color, and flavor. Samples (10 g of IML samples) were identified using a three-digit random number and served following the randomized complete block design (RCBD).

### 2.5. Accelerated Shelf Life Testing Procedure and Kinetics Calculations

To determine the reaction order, zero and first-order equations were applied separately, and the instrumental color values and consumer acceptance scores of color were plotted as a function of storage time. Thus, the linear regression and exponential plots were carried out for these values, corresponding to the values of *k* (reaction velocity) by zero (Equation (1)) and first- (Equation (2)) order equations for each temperature [21].

(1) $$Q=Q_{o}-kt$$

(2) $$ln\;\frac{Q}{Q_{o}}=-kt$$

where *Q*_0_ represents the initial value of a quality attribute and *Q* is the value of that attribute at time *t.*

As the quality parameters followed zero or first-order reactions, Equations (3) and (4) were used to predict the time (*t_s_*) that is needed for changes in color and consumer acceptance scores of color indices [21].

(3) $$t_{s}=\frac{Q_{o\;\;}-Q_{e}\;}{k}$$

(4) $$t_{s}=\frac{ln\frac{Q_{o}}{Q_{e}}\;}{k}$$

where *t_s_* is the time predicted for these changes to happen. The acceptance threshold (*Q_e_*) or the reject point was when the consumer acceptance score in this shelf life study was less than 5.

### 2.6. Water Vapor Permeability of Bags

Water vapor permeability of the Al bag and clear plastic bag were determined using a Water Vapor Transmission Rate Tester (C303H, LABTHINK, Jinan, China) at a temperature of 38 °C and 50% relative humidity (RH). Two replicates were measured for each sample.

### 2.7. Statistical Analysis

A factorial design experiment in completely randomized design was conducted in this study. The main factors were different storage temperatures (4, 25, 35 and 45 °C). A temperature of 4 °C acts as a stable control for the baseline comparison, 25 °C simulates the real-world ambient storage condition, and the elevated temperatures (35 °C and 45 °C) accelerate degradation to rapidly predict shelf life and test the product’s stability under extreme abuse. Three different types of packaging (Al bag with nitrogen, Al bag without nitrogen and clear plastic bag) were used. Analysis of variance (ANOVA) was carried out by using the SPSS version 16.0.

## 3. Results and Discussion

### 3.1. Quality Changes in IML During Storage Time

To estimate the quality changes in IML products during storage time, the IML products were kept in three different kinds of packaging, including an Al bag with nitrogen, an Al bag without nitrogen, and a clear plastic bag, and stored at different temperatures (4, 25, 35, and 45 °C) for 24 weeks. Overall, the temperatures of storage and packaging types were the major factors that influenced the changes in physical, chemical, and sensory properties of IML samples during storage.

#### 3.1.1. Water Activity (a_w_) and Moisture Contents (MCs)

**Figure 1:**
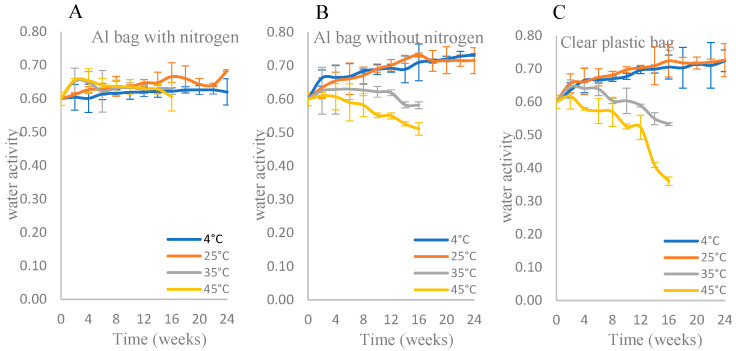
Water activities (a_w_) of IML packed in different packaging types: (**A**) Al bag with nitrogen, (**B**) Al bag without nitrogen, and (**C**) clear plastic bag during storage time at different temperatures (4, 25, 35, and 45 °C). See Supplementary Tables S1–S3 for mean and SD values.

**Figure 2:**
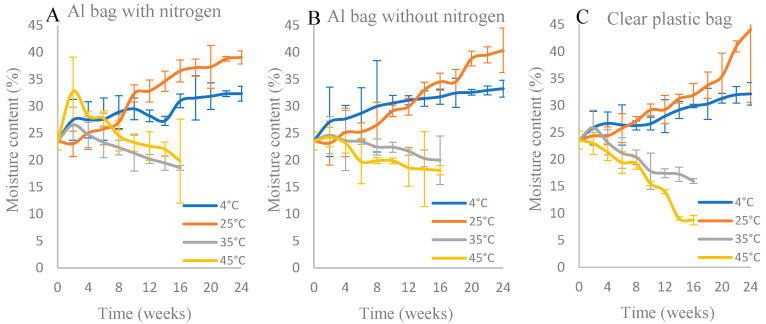
Moisture contents (% w.b.) of IML packed in different packaging types: (**A**) Al bag with nitrogen, (**B**) Al bag without nitrogen, and (**C**) clear plastic bag during storage time at different temperatures (4, 25, 35, and 45 °C). See Supplementary Tables S4–S6 for mean and SD values.

The a_w_ and MC of the IML samples are shown in Figure 1 and Figure 2. From the ANOVA results (see Supplementary Materials), the temperatures of storage and packaging types were significant factors affecting the water activity and moisture content of longan samples (*p* ≤ 0.05). Regardless of the storage temperatures, the IML samples packed in the Al bag with nitrogen maintained an a_w_ between 0.6 and 0.7 (Figure 1). In contrast, the a_w_ of the samples stored in clear plastic bags at 35 and 45 °C drastically dropped over time, while for those stored at 4 and 25 °C, the a_w_ gradually increased and reached a value of 0.72 after 24 weeks of storage (Figure 1). The a_w_ values of the samples packed in an Al bag without nitrogen or a clear plastic bag stored at 35 and 45 °C were not measured after 16 weeks due to unacceptable consumer acceptance scores. Similar trends were observed for the changes in MC (Figure 2), except for the effects of storage temperatures on MC for the samples packed in Al with nitrogen. The initial MC of samples after processing was 23.58 ± 1.68% (w.b.). The MC of the samples stored at 4 or 25 °C increased, while it decreased for those stored at 35 and 45 °C. (Figure 2). Without exception, the MCs of all samples stored at 35 and 45 °C were not measured after 16 weeks (Figure 2) due to unacceptable consumer acceptance scores.

According to the ANOVA results, the storage temperature and packaging type within the storage period significantly (*p* ≤ 0.05) affected the a_w_ and MC of IML samples. A similar observation was reported by Panwar et al. [34] for the intermediate moisture aonla (*Phyllanthus emblica* L.) segments, which were packed in low–density polyethylene bags and stored at room temperature for 6 months. Regardless of the packaging types, the a_w_ and MC of IML samples stored at 35 °C and 45 °C tended to decrease during 16 weeks of storage due to the phenomenon of evaporation occurring inside these packages. Aluminum is known to provide good gas and moisture barrier properties, especially when used with nitrogen [35]. The WVP of the clear polyamide bag was 4.816 × 10^−15^ ± 0.014 g·cm/(cm^2^·Pa s), while that of the Al bag was 1.964 × 10^−17^ ± 0.121 g·cm/(cm^2^·Pa s). As expected, the Al bag with nitrogen could retain MC better than the others (Figure 2).

Depending on the storage temperature, IMLs packed in a clear plastic bag gained or lost moisture content due to the higher water vapor permeability (WVP) of this bag compared with the Al bag. An increase in moisture content of IML during storage at 4 °C and 25 °C was observed. This might be attributed to the internal moisture migration and redistribution driven by the temperature gradients [36]. The equilibrium moisture content of IML is temperature-dependent and increases as the temperature decreases. The product’s capacity to hold water increases at lower temperatures, leading to the absorption of vapor from the package headspace. The higher WVP of the clear plastic bag compared to that of the aluminum-laminated bag also facilitated the moisture transfer from the external environment, hence a higher moisture content. This underscores that for IML, packaging permeability can influence product stability, not only by preventing moisture loss but also by modulating moisture gain under specific temperature–humidity conditions.

#### 3.1.2. Microbial Analysis

There was no microorganism growth until the 16th week, 12th week, and 8th week for IML products packed into the Al bag with nitrogen, Al bag without nitrogen, and clear plastic bag and stored at 4 °C, respectively. Correspondingly, these growths increased at the 24th week, 16th week, and 12th week until the end of storage, respectively. As the shelf life of IML products packed in three different types packaging and stored at 35 °C and 45 °C ended at the 20th week due to unacceptable consumer acceptance scores (<5.0), the microbial analyses were not performed. According to the Microbiological Guidelines for Food [35], the satisfactory total number of bacteria in an aerobic environment in moderate temperature, i.e., “Aerobic colony count,” for sweet foods is <10^4^ cfu/g products. In this study, the IML samples stored at 4 °C and 25 °C had a TBC lower than the standard (10^4^ cfu/g). In addition, it could clearly be seen that YMC and E. coli were not detected in all samples at all storage temperatures over a period 6 months in this study (Table 1).

The combination of the OD drying method with packaging conditions improved the microbial stability and delayed the growth of microbes. The reduction in microbial growth and the improved shelf life can be attributed not solely to osmotic dehydration, but also to the preservative action of potassium sorbate. Saxena et al. [37] reported that TBC and YMC can be detected in intermediate moisture pineapple (a_w_ = 0.82) packed in high-density polyethylene bags and stored at ambient temperature at 2 × 10^2^ cfu/g at 20 days of storage. Furthermore, Chaturvedi et al. [38] found the TBC and YMC in intermediate moisture carrot (a_w_ = 0.6) packed in polyethylene bags and stored at ambient temperature to be 2.1 × 10^2^ cfu/g and 2.08 × 10^2^ cfu/g, respectively. In this study, a_w_ of IML packed in the Al bag with nitrogen was in the range of 0.6–0.7 for the whole storage time at 4–45 °C. The highest value of TBC (6.5 × 10 cfu/g) and YMC (<2.5 × 10^−1^ cfu/g) were found after storage for 24 weeks. These values are significantly lower than those reported by Chaturvedi et al., likely due to the limited oxygen in the Al bag with nitrogen compared with the polyethylene bag, which was more oxygen-permeable. The a_w_ is an important parameter that can affect microbial count and is important for the shelf life of intermediate moisture products [8]. Homhuan [39] reported that yeast and mold were not detected in dried longan, which was produced using OD and packed in aluminum foil-laminated plastic bags with nitrogen. Moreover, the Microbiological Guidelines for Food [35] state that *E. coli* is a commonly used fecal indicator organism. Its presence in food generally indicates direct or indirect fecal contamination. A substantial number of *E. coli* in food implies a general lack of cleanliness in handling and improper storage. The satisfactory total number of *E. coli* present in food is <20 cfu/g. In this study, the IML products were safe for human consumption as *E. coli* was absent. In general, the microbial counts were only detected at the 24th week for IML packed in an Al bag with nitrogen, at the 16th week for IML packed in an Al bag without nitrogen and at the 12th week for IML packed in clear plastic bag stored at 4 °C over a period of 6 months of storage. In fact, nitrogen is a useful filler gas to prevent package collapse caused by carbon dioxide dissolving in the food [40]. Carbon dioxide inhibits the growth of many spoilage bacteria, and nitrogen indirectly helps to slow down the growth of aerobic spoilage microbes in unpreserved foods [40]. Therefore, in this study, when IML products were packed in an Al bag with nitrogen, the microbial safety was enhanced and most effective, followed by IML products packed in an Al bag without nitrogen and a clear plastic bag. Finally, IML products packed in an Al bag with nitrogen could be kept longer than the others.

#### 3.1.3. Color Analysis

**Figure 3:**
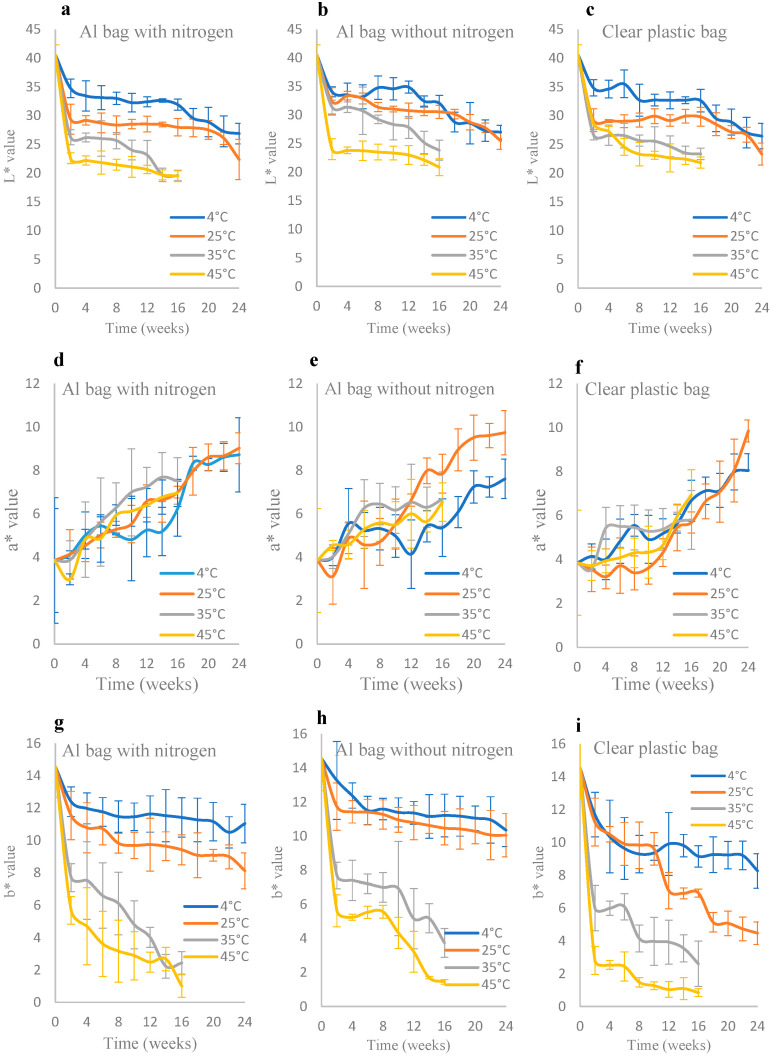
Color changes in IML packed in different packaging materials during storage time at different temperatures (4, 25, 35, and 45 °C), expressed as L* (**a**–**c**), a* (**d**–**f**), and b* (**g**–**i**) values of IML packed in three different packing materials.

Color changes in IML samples during storage can clearly be seen in Figure 3. The ANOVA results showed that storage temperature and packaging type within the storage period significantly (*p* ≤ 0.05) affected the color values of IML (see Supplementary Materials). During storage, L* and b* values generally decreased (darker and less yellow color), while the a* value increased (redder color). Temperature was the most critical factor accelerating color changes. This was due to the increased rates of chemical reactions like Maillard browning and non-enzymatic oxidation. ΔE values of IML stored at 4 °C increased gradually, reaching a maximum of ~15.0–15.7 after 24 weeks. At 25 °C, ΔE values were significantly higher than at 4 °C, reaching up to ~16.6–21.7 after 24 weeks. The clear plastic bag showed the most drastic increase. At 35 °C and 45 °C, the browning was rapid and severe. ΔE climbed to extremely high levels (19.6–24.0) by week 16, representing an obvious color change that would make the product unacceptable to consumers. The Al bag with nitrogen provided the best protection. The nitrogen flush displaced oxygen, drastically slowing down oxidative reactions. The initial L* value was 40.6 ± 1.73. After 16 weeks of storage, the L* values of IML in the aluminum foil-laminated bags with nitrogen at 4, 25, 35, and 45 °C decreased to 31.92 ± 0.97, 27.99 ± 1.53, 19.55 ± 0.89, and 19.54 ± 1.00, respectively. Overall, storage at 4 °C maintained the color of IML samples more effectively than at other storage temperatures.

Color is a very essential quality characteristic of fruit and vegetable products because it influences consumer acceptability [41]. The browning reaction in this study was non-enzymatic browning reaction, possibly a Maillard reaction and ascorbic acid oxidation, because the longan contained reducing sugar, amino acids, and ascorbic acid. The Maillard reaction is caused by the interaction between free sugar content and free amino acid content in longan fruits during drying [42,43]. The amount of free sugar in the longan flesh is presented in the order of glucose, maltose, sucrose, xylose, and fructose, while the quantities of some of the amino acids, such as proline and leucine, significantly decrease after drying [43]. Browning in dried longan fruit during storage is primarily caused by the Maillard reaction, a form of non-enzymatic browning that requires oxygen and occurs most rapidly at an a_w_ of between 0.6 and 0.7. It can be delayed by storing the product at low temperatures and avoiding oxygen by nitrogen flushing to replace the oxygen inside the package [44]. Therefore, the use of an Al bag with nitrogen, which displaces oxygen with an inert nitrogen, effectively slows the progression of the Maillard reaction. Moreover, the rate of the Maillard reaction increased as the heating temperature increased [45]. Therefore, L* and b* values of IML stored at 35 °C and 45 °C decreased faster than those of IML stored at 4 °C and 25 °C. Moreover, Wangchareon [46] found that the Maillard reaction could occur between carbonyl groups and amino compounds, while ascorbic acid oxidation could be the thermal decomposition of ascorbic acid. Wall [44] reported that longan fruit had the highest ascorbic acid content (60.1 mg/100 g fresh weight) among the three fruits, longan, lychee, and rambutan.

#### 3.1.4. Textural Analysis

**Figure 4:**
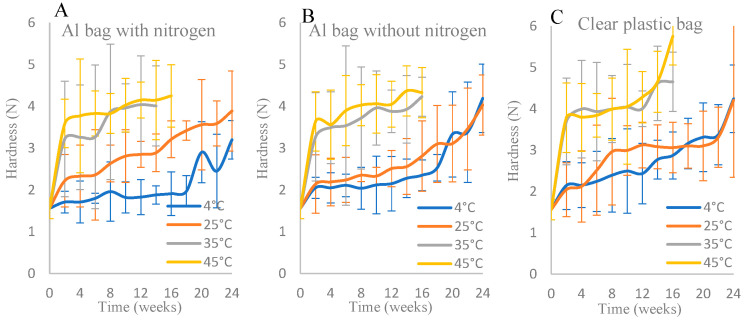
Texture analysis of IML packed in different packages: (**A**) Al bag with nitrogen, (**B**) Al bag without nitrogen, and (**C**) clear plastic bag during storage times at different temperatures (4, 25, 35, and 45 °C).

Figure 4 shows the changes in texture hardness (N) of IML samples stored in different packaging and storage temperature over a period of 6 months. According to the ANOVA results (see Supplementary Materials), the effects of storage temperature and packaging within the storage period on the texture of IML samples were significant (*p* ≤ 0.05). In general, the effect of the interaction between storage temperature and packaging within the storage period on the hardness characteristic of IML samples was not significant (*p* > 0.05). The hardness of the intermediate moisture longan (IML) increased over time during storage. At higher storage temperatures (35 °C and 45 °C), the hardness increased drastically during the first 8 weeks. In addition, the packaging type affected the hardness. IML packed in the aluminum bags, with and without nitrogen, was softer than that packed in the clear plastic bags due to the better moisture protection offered by the aluminum film.

Robertson [47] stated that changes in a_w_ could either inhibit or promote a physical change in the nature of the sugar present in food products, which, in turn, affects the texture. A low a_w_ maintains the sugar in an amorphous form in food products [43]. This amorphous sugar matrix undergoes glass transition [48], contributing to the texture hardening of IML. A decrease in water activity (a_w_) raises the glass transition temperature (Tg) because water acts as a plasticizer; less water reduces molecular mobility and promotes a glassy, rigid structure. This transition limits diffusion and increases the firmness of the product [49,50,51]. In this study, the texture of IML samples stored at high temperatures was harder than that of samples stored at low temperatures, because the a_w_ of the former IML samples was lower than that of the latter samples (Figure 1 and Figure 4). The hardness of normal dried longan dried in a hot air oven has been found to be 9.76 ± 1.56 (N) and 6.71 ± 1.79 (N) [18,49]. In this study, however, the texture of IML products stored in three different packaging materials was softer than that of normal dried longan. Generally, the hardness of IML products packed in an Al bag with nitrogen was softer than that of IML products packed in an Al bag without nitrogen and a clear plastic bag because aluminum foil provides high reflectivity and high conductivity [43], which can affect the temperature of the food, hence affecting the texture of the food. Therefore, IML products packed in an Al bag with nitrogen could be kept longer than those packed in the other types of packaging used in this study.

#### 3.1.5. Consumer Acceptance

Consumers gave overall acceptance scores ranging from 3.3 (close to dislike moderately) to 6.6 (close to like moderately) (Table 2). The effects of interaction between storage temperature and packaging on the overall acceptability, odor, and color of IML samples were significant (*p* ≤ 0.05). The sensorial scores of IML at the beginning period were 6.2 ± 1.4 for overall liking, 6.3 ± 1.5 for color testing, and 6.0 ± 1.7 for flavor testing. It can be seen in Table 2 that IML packed in an Al bag with and without nitrogen and clear plastic bags at 4 °C were acceptable during storage time.

In shelf life testing, consumer acceptance could be a determining factor with a testing score of above 5.0 [52]. A study to select the optimal formulation of strawberry–longan bars used acceptability scores of at least 5.6 for appearance and texture [53]. Grosso & Resurreccion [54] reported that on the 9-point hedonic scale, a food sample is considered “neither liked nor disliked” if its overall acceptability score is 5. If a food sample has a value of 4, it means “dislike slightly.” Therefore, values below 5 on the 9-point hedonic scale might be regarded as a threshold for determining if a food item is unsatisfactory to the consumer. In this current study, the shelf life of the IML samples was ended when the sensorial acceptance score was below 5.0. The acceptance scores of IML products packed in the Al bag with nitrogen were higher than those of IML products packed in the Al bag without nitrogen and clear plastic bag during storage. Therefore, the qualities of IML products packed in the Al bag with nitrogen could be maintained longer than those of products packed in other packaging materials.

### 3.2. Shelf Life Prediction of Intermediate Moisture Longan

**Figure 5:**
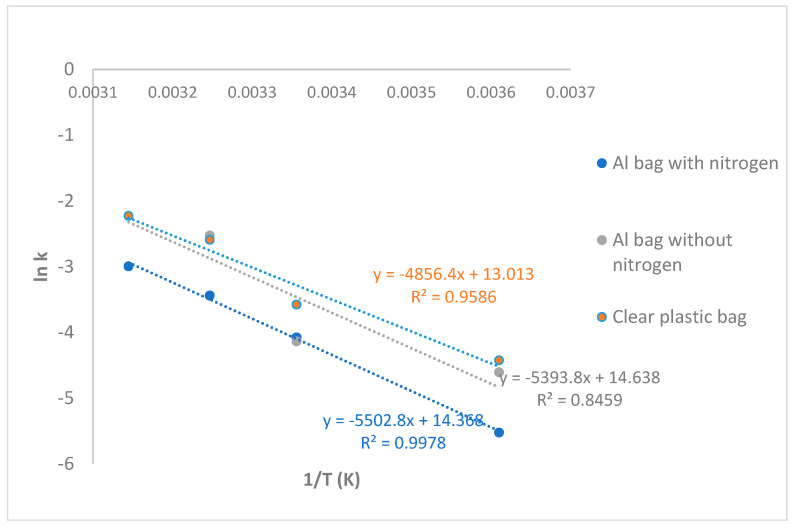
Arrhenius plot of IML stored at 4 °C, 25 °C, 35 °C, and 45 °C in Al bag with nitrogen, Al bag without nitrogen, and clear plastic bag.

The quality changes in IML samples packed and stored in different conditions were clearly observed, especially the changes in color of IML products. These changes were significant according to the storage conditions of storage temperatures and packaging types (*p* ≤ 0.05). The L* values of IML products continued to decline during storage, which resulted in declined consumer acceptance scores of color. The rejection point for sensory evaluation in this study is a consumer acceptance score of less than 5.0. The linear plot illustrated that the rates of color values and color acceptance changes were constant throughout the storage period, demonstrating the zero order reaction, and the exponential plot between color acceptance scores and storage time represented the first-order reactions. Table 3 and Table 4 show the constant rates of color changes (L* values) and color acceptance changes in IMLs during storage, in which IMLs were packed into an Al bag with nitrogen, an Al bag without nitrogen, and a clear plastic bag and stored at 4, 25, 35, and 45 °C. The kinetics of color change were analyzed using the L* value (lightness) and the consumer color acceptance score. L* values were measured every two weeks for 24 weeks at 4 °C and 25 °C, and for 16 weeks at 35 °C and 45 °C. The color acceptance tests were conducted for up to 24 weeks, or until the product was no longer deemed acceptable by consumers. The accuracy of the rate constant (k) is dependent on the number of data points; a greater number of data points yields a more accurate estimation of k. Based on Table 3 and Table 4, the first-order model with a higher R^2^ was determined to be more appropriate and was employed for the shelf life prediction. The Arrhenius plot was used to describe how the rate of a chemical reaction depended on storage temperature (Figure 5). It quantitatively showed that the rate of color change increased exponentially as the temperature increased. The rate constant, k, determined the reaction rate. According to this experiment, k values were higher at higher temperatures (35 and 45 °C) than at lower temperatures (25 and 4 °C) in all packaging types. Generally, the first-order reactions could be used to describe the changes in these attributes. The low values of rate constants for all quality parameters for an Al bag with nitrogen in comparison to those for an Al bag without nitrogen and a clear plastic bag indicated that less changes occurred in IML products when they were packed in Al bag with nitrogen compared to the others. It illustrated that the Al bag with nitrogen provided better protection than the other materials. The activation energy (Ea) of IML packed in the Al bag with nitrogen, the Al bag without nitrogen, and the clear plastic bag were 45.74 kJ/mol, 44.85 kJ/mol, and 40.38 kJ/mol, respectively. In addition, the regression equations can be used to estimate the shelf life of IML products by determining the k value at desired temperatures. The shelf life of IML products can be judged via the changes in color acceptability in sensory evaluation. The acceptance score of color was found to be 6.3 at the beginning of storage, and the reject point was 5.0 for IML products. From the results of this study, the stable shelf life of IML products packed into three packages at 4 °C, followed by at 25 °C, could provide good quality of color and high scores of sensory acceptability. The experimental shelf life and predicted shelf life of IML stored at 4 °C, 25 °C, 35 °C, and 45 °C are reported in Table 5. The experimental shelf life of IML in an Al bag with nitrogen at 4 °C, 25 °C, 35 °C, and 45 °C was >24, 15, 12, and 3 weeks, respectively. While the predicted shelf life of IML in an Al bag with nitrogen at 4 °C, 25 °C, 35 °C, and 45 °C was 58, 14, 7, and 4 weeks, respectively. A direct comparison between the experimentally observed shelf life and the model-predicted shelf life is presented in Table 5. The model demonstrated high accuracy at temperatures of 25 °C and above. Notably, for samples stored at 4 °C, the model predicted a shelf life of 58 weeks for IML packaged in aluminum bag with nitrogen. This value was a model extrapolation, as the experimental observation was terminated at 24 weeks, at which point the product acceptance score of 5.6 (Table 2) still exceeded the minimum acceptability score (a value of 5.0). Therefore, the experimentally confirmed shelf life at 4 °C was at least 24 weeks, while the Arrhenius model provided a theoretical projection of 58 weeks.

The core discrepancy arises because the product’s failure mechanism at 4 °C differs from the higher temperatures used to build the Arrhenius model. At 25-45 °C, spoilage is dominated by rapid chemical reactions like Maillard browning, which are accurately captured by the model. However, the aluminum bag with nitrogen effectively suppresses these reactions at 4 °C. Consequently, the primary mode of failure shifts to slower, less temperature-sensitive processes. This highlights a key limitation of accelerated shelf life studies: the assumption that the same reaction causes failure at all temperatures. Our prediction at 4 °C might deviate from this core ASLS assumption, specifically for the nitrogen-flushed aluminum packaging. Furthermore, predicting 58 weeks is a significant extrapolation from collecting data. Any minor error in the model’s activation energy is greatly magnified in the predicted shelf life. Therefore, while the model is accurate at higher temperatures, its prediction at 4 °C is limited by this change in the failure mechanism.

The previous studies reported that the storage quality of osmotically dehydrated papaya products had remained for up to 6 months at an ambient temperature [8], and banana products were stored up to one year or more, depending on the storage conditions and packaging materials used [55]. In a study by Akharume et al. [56], osmotically dehydrated apples were stored in non-vacuum and vacuum polyethylene packaging. Vacuum packaging was found to improve the stability of both untreated and sugar-infused dried apples at room temperature. However, after 120 days (~17 weeks), the total bacterial count (TBC) for their samples remained between 10^3^ and 10^4^ CFU/g (log 3–4). In contrast, all packaging treatments in our study, particularly the Al bag with nitrogen, achieved lower TBC values of 10^1^–10^2^ CFU/g (log 1–2) after 20 weeks. The superior microbial inhibition in our study was attributed to the oxygen-free environment created by nitrogen flushing in the Al bag, compared to the packages where oxygen was present (the Al bag without nitrogen and the clear plastic bag). Furthermore, the aluminum bags provide a better barrier against oxygen ingress during storage. This was demonstrated by the oxygen permeability values, which were 0.10 ± 0.004 cc/m^2^·day for the aluminum bags and 95.9 ± 1.4 cc/m^2^·day for the clear plastic bags (LLDPE/nylon). Furthermore, Jimeno et al. [57] studied that the intermediate moisture mango roll could be packed in aluminum foil with polyethylene (PE) liner and stored for 9 months without any change in a_w_ or product color. In this study, the Al bag with nitrogen was more effective in maintaining the quality of the IML samples than the other packaging, and the storage temperature of 4 °C was most effective in maintaining the quality of the IML.

## 4. Conclusions

The application of multiple preservation hurdles, namely osmotic dehydration, hot-air drying, packaging type, and storage temperature, was shown to effectively extend the shelf life of IML to 14, 14, and 8 weeks (based on the ASLT) when stored in the aluminum (Al) bag with nitrogen, the Al bag without nitrogen, and the clear plastic bag at 25 °C, respectively. Throughout storage, significant variations (*p* ≤ 0.05) in moisture content, color, and textural attributes were observed as a function of both storage temperature and packaging type. In addition, osmotic dehydration combined with hot-air drying effectively reduced water activity (a_w_), thereby suppressing microbial growth and maintaining the sensory acceptability of IML products over the entire storage period. The progression of quality changes followed the first-order reaction kinetics, with kinetic rate constants for IML packaged in Al bags with nitrogen (at 25 °C; for L*: k = 1.7 × 10^−2^ and for acceptance score: k = 0.8 × 10^−2^) being lower than those of samples stored in the Al bags without nitrogen (for L*: k = 1.8 × 10^−2^ and for acceptance score: k = 1.7 × 10^−2^) and the clear plastic bags (for L*: k = 1.9 × 10^−2^ and for acceptance score: k = 1.8 × 10^−2^). These findings indicated that the use of Al bags with nitrogen is the most effective strategy for prolonging the shelf life of IML products while minimizing quality deterioration. Future development should focus on optimizing this hurdle technology for a wider range of fruits and vegetables and exploring more sustainable, high-barrier packaging alternatives to aluminum.

## Figures and Tables

**Table 1:** Microbial analysis of IML samples packed in different packages at different temperatures (4, 25, 35, and 45 °C) during 6 months of storage.

Storage (Weeks)		Al Bag with Nitrogen				Al Bag without Nitrogen				Clear Plastic Bag			
		4 °C	25 °C	35 °C	45 °C	4 °C	25 °C	35 °C	45 °C	4 °C	25 °C	35 °C	45 °C
Total Bacterial Count (cfu/g)	0	<2.5 × 10^−1^	<2.5 × 10^−1^	<2.5 × 10^−1^	<2.5 × 10^−1^	<2.5 × 10^−1^	<2.5 × 10^−1^	<2.5 × 10^−1^	<2.5 × 10^−1^	<2.5 × 10^−1^	<2.5 × 10^−1^	<2.5 × 10^−1^	<2.5 × 10^−1^
	4	<2.5 × 10^−1^	<2.5 × 10^−1^	<2.5 × 10^−1^	<2.5 × 10^−1^	<2.5 × 10^−1^	<2.5 × 10^−1^	<2.5 × 10^−1^	<2.5 × 10^−1^	<2.5 × 10^−1^	<2.5 × 10^−1^	<2.5 × 10^−1^	<2.5 × 10^−1^
	8	<2.5 × 10^−1^	<2.5 × 10^−1^	<2.5 × 10^−1^	<2.5 × 10^−1^	<2.5 × 10^−1^	<2.5 × 10^−1^	<2.5 × 10^−1^	<2.5 × 10^−1^	<2.5 × 10^−1^	<2.5 × 10^−1^	<2.5 × 10^−1^	<2.5 × 10^−1^
	12	<2.5 × 10^−1^	<2.5 × 10^−1^	<2.5 × 10^−1^	<2.5 × 10^−1^	<2.5 × 10^−1^	<2.5 × 10^−1^	<2.5 × 10^−1^	<2.5 × 10^−1^	40 ± 4	40 ± 6	<2.5 × 10^−1^	<2.5 × 10^−1^
	16	<2.5 × 10^−1^	<2.5 × 10^−1^	<2.5 × 10^−1^	<2.5 × 10^−1^	55 ± 4	25 ± 3	20 ± 2	20 ± 4	30 ± 5	20 ± 4	45 ± 6	50 ± 10
	20	<2.5 × 10^−1^	<2.5 × 10^−1^	-	-	55 ± 2	60 ± 4	-	-	45 ± 3	30 ± 2	-	-
	24	65 ± 2	65 ± 8	-	-	75 ± 11	85± 9	-	-	50 ± 5	55 ± 5	-	-
Yeast and Mold Count (cfu/g)	0	<2.5 × 10^−1^	<2.5 × 10^−1^	<2.5 × 10^−1^	<2.5 × 10^−1^	<2.5 × 10^−1^	<2.5 × 10^−1^	<2.5 × 10^−1^	<2.5 × 10^−1^	<2.5 × 10^−1^	<2.5 × 10^−1^	<2.5 × 10^−1^	<2.5 × 10^−1^
	4	<2.5 × 10^−1^	<2.5 × 10^−1^	<2.5 × 10^−1^	<2.5 × 10^−1^	<2.5 × 10^−1^	<2.5 × 10^−1^	<2.5 × 10^−1^	<2.5 × 10^−1^	<2.5 × 10^−1^	<2.5 × 10^−1^	<2.5 × 10^−1^	<2.5 × 10^−1^
	8	<2.5 × 10^−1^	<2.5 × 10^−1^	<2.5 × 10^−1^	<2.5 × 10^−1^	<2.5 × 10^−1^	<2.5 × 10^−1^	<2.5 × 10^−1^	<2.5 × 10^−1^	<2.5 × 10^−1^	<2.5 × 10^−1^	<2.5 × 10^−1^	<2.5 × 10^−1^
	12	<2.5 × 10^−1^	<2.5 × 10^−1^	<2.5 × 10^−1^	<2.5 × 10^−1^	<2.5 × 10^−1^	<2.5 × 10^−1^	<2.5 × 10^−1^	<2.5 × 10^−1^	<2.5 × 10^−1^	<2.5 × 10^−1^	<2.5 × 10^−1^	<2.5 × 10^−1^
	16	<2.5 × 10^−1^	<2.5 × 10^−1^	<2.5 × 10^−1^	<2.5 × 10^−1^	<2.5 × 10^−1^	<2.5 × 10^−1^	<2.5 × 10^−1^	<2.5 × 10^−1^	<2.5 × 10^−1^	<2.5 × 10^−1^	<2.5 × 10^−1^	<2.5 × 10^−1^
	20	<2.5 × 10^−1^	<2.5 × 10^−1^	−	−	<2.5 × 10^−1^	<2.5 × 10^−1^	−	−	<2.5 × 10^−1^	<2.5 × 10^−1^	−	−
	24	<2.5 × 10^−1^	<2.5 × 10^−1^	−	−	<2.5 × 10^−1^	<2.5 × 10^−1^	−	−	<2.5 × 10^−1^	<2.5 × 10^−1^	−	−

**Table 2:** Consumer acceptance scores ^1^ of IML samples packed in different packages at different temperatures (4, 25, 35, and 45 °C) during 6 months of storage.

Storage (Weeks)		Al Bag with Nitrogen				Al Bag Without Nitrogen				Clear Plastic Bag			
		4 °C	25 °C	35 °C	45 °C	4 °C	25 °C	35 °C	45 °C	4 °C	25 °C	35 °C	45 °C
Overall acceptance	3	6.4 ± 1.3	6.2 ± 1.5	5.9 ± 1.5	5.7 ± 1.5	6.3 ± 1.4	5.9 ± 1.4	4.8 ± 1.4	4.1 ± 1.5	6.4 ± 1.3	5.7 ± 1.6	5.2 ± 1.4	4.5 ± 1.6
	6	6.5 ± 1.5	6.3 ± 1.6	5.7 ± 1.6	5.0 ± 1.6	6.5 ± 1.3	5.7 ± 1.3	4.5 ± 1.7	3.9 ± 1.9	6.4 ± 1.3	5.7 ± 1.3	4.8 ± 1.6	4.1 ± 1.9
	9	6.2 ± 1.6	5.8 ± 1.3	5.4 ± 1.5	4.3 ± 1.9	6.2 ± 1.5	5.4 ± 1.2	-	-	6.2 ± 1.4	5.4 ± 1.3	4.3 ± 1.7	-
	12	6.3 ± 1.5	5.8 ± 1.4	5.1 ± 1.6	4.0 ± 1.7	6.1 ± 1.5	5.6 ± 1.3	-	-	6.2 ± 1.6	4.9 ± 1.3	-	-
	15	6.3 ± 1.5	5.3 ± 1.4	4.1 ± 1.8	-	6.2 ± 1.4	5.1 ± 1.5	-	-	6.4 ± 1.5	4.8 ± 1.5	-	-
	18	6.3 ± 1.5	4.9 ± 1.3	-	-	5.4 ± 1.2	4.7 ± 1.1	-	-	5.7 ± 1.4	-	-	-
	21	6.1 ± 1.3	- ^2^	-	-	5.3 ± 1.1	-	-	-	5.5 ± 1.2	-	-	-
	24	5.6 ± 1.3	-	-	-	4.9 ± 1.1	-	-	-	4.6 ± 1.3	-	-	-
Color	3	6.6 ± 1.3	6.2 ± 1.4	6.1 ± 1.6	5.6 ± 1.7	6.4 ± 1.3	6.0 ± 1.3	4.8 ± 1.6	3.5 ± 1.8	6.6 ± 1.6	5.9 ± 1.6	4.5 ± 1.5	3.6 ± 1.8
	6	6.5 ± 1.3	6.2 ± 1.2	5.9 ± 1.6	4.9 ± 1.7	6.5 ± 1.4	5.8 ± 1.4	4.4 ± 1.9	3.3 ± 1.9	6.6 ± 1.5	5.2 ± 1.5	4.6 ± 1.5	3.3 ± 1.6
	9	6.5 ± 1.6	6.1 ± 1.4	5.4 ± 1.5	4.1 ± 1.7	6.1 ± 1.5	5.5 ± 1.2	-	-	6.2 ± 1.6	5.5 ± 1.4	4.3 ± 1.7	-
	12	6.2 ± 1.1	6.1 ± 1.5	5.1 ± 1.8	-	6.5 ± 1.6	5.6 ± 1.4.	-	-	6.5 ± 1.5	4.9 ± 1.2	-	-
	15	6.3 ± 1.0	5.6 ± 1.4	4.1 ± 1.9	-	6.3 ± 1.4	5.2 ± 1.6	-	-	6.6 ± 1.5	4.8 ± 1.5	-	-
	18	6.1 ± 1.1	4.7 ± 1.2	-	-	5.8 ± 1.2	4.8 ± 1.3	-	-	5.9 ± 1.3	-	-	-
	21	6.0 ± 1.0	-	-	-	5.7 ± 1.2	-	-	-	5.6 ± 1.1	-	-	-
	24	5.8 ± 1.1	-	-	-	5.0 ± 1.2	-	-	-	4.7 ± 1.3	-	-	-
Flavor	3	6.0 ± 1.6	6.0 ± 1.7	5.8 ± 1.5	5.5 ± 1.7	6.1 ± 1.5	6.1 ± 1.5	4.8 ± 1.6	4.5 ± 1.8	6.0 ± 1.5	5.6 ± 1.5	5.2 ± 1.7	4.6 ± 1.5
	6	6.1 ± 1.6	5.8 ± 1.7	5.6 ± 1.6	5.1 ± 1.6	6.0 ± 1.5	5.8 ± 1.7	4.7 ± 1.7	4.2 ± 1.7	6.0 ± 1.4	5.5 ± 1.5	5.0 ± 1.7	4.5 ± 1.8
	9	6.0 ± 1.8	5.4 ± 1.4	5.1 ± 1.4	4.4 ± 1.8	5.7 ± 1.4	5.2 ± 1.3	-	-	5.9 ± 1.3	5.4 ± 1.4	4.7 ± 1.7	-
	12	6.0 ± 1.5	5.5 ± 1.6	4.8 ± 1.6	4.1 ± 1.7	5.8 ± 1.5	5.5 ± 1.5	-	-	5.9 ± 1.5	5.1 ± 1.2	4.5 ± 1.6	-
	15	6.0 ± 1.6	5.2 ± 1.5	3.9 ± 1.7	-	6.1 ± 1.6	5.0 ± 1.6	-	-	6.1 ± 1.5	4.7 ± 1.4	-	-
	18	5.9 ± 1.7	4.9 ± 1.5	-	-	5.6 ± 1.4	4.7 ± 1.3	-	-	5.7 ± 1.3	4.6 ± 1.3	-	-
	21	5.8 ± 1.5	-	-	-	5.4 ± 1.2	-	-	-	5.5 ± 1.2	-	-	-
	24	5.4 ± 1.3	-	-	-	-	-	-	-	4.8 ± 1.4	-	-	-

^1^ Based on a 9-point hedonic scale. N = 50 consumers. ^2^—no measurement because shelf life was ended.

**Table 3:** Kinetic constants of L* value changes in IML products packed in different packages and stored at 4, 25, 35, and 45 °C during storage.

Reaction Order	°C	Al Bag with Nitrogen		Al Bag Without Nitrogen		Clear Plastic Bag	
		Constant Rate (k)	R^2^	Constant Rate (k)	R^2^	Constant Rate (k)	R^2^
Zero order	4	4.5 × 10^−1^	0.864	4.3 × 10^−1^	0.782	4.6 × 10^−1^	0.878
	25	3.7 × 10^−1^	0.529	4.3 × 10^−1^	0.831	3.4 × 10^−1^	0.482
	35	0.1 × 10^1^	0.764	7.9 × 10^−1^	0.813	6.9 × 10^−1^	0.525
	45	8.1 × 10^−1^	0.452	7.2 × 10^−1^	0.444	8.7 × 10^−1^	0.648
First-order	4	1.3 × 10^−2^	0.868	1.7 × 10^−2^	0.982	1.5 × 10^−2^	0.939
	25	1.7 × 10^−2^	0.768	1.8 × 10^−2^	0.946	1.9 × 10^−2^	0.904
	35	4.5 × 10^−2^	0.980	3.2 × 10^−2^	0.976	3.3 × 10^−2^	0.835
	45	4.9 × 10^−2^	0.972	4.2 × 10^−2^	0.955	3.5 × 10^−2^	0.755

**Table 4:** Kinetic constants of color acceptance score changes in IML products packed in different packages and stored at 4, 25, 35, and 45 °C during storage.

Reaction Order	°C	Al Bag with Nitrogen		Al Bag Without Nitrogen		Clear Plastic Bag	
		Constant Rate (k)	R^2^	Constant Rate (k)	R^2^	Constant Rate (k)	R^2^
Zero order	4	2.7 × 10^−2^	0.708	5.0 × 10^−2^	0.810	5.8 × 10^−2^	0.747
	25	7.6 × 10^−2^	0.682	8.6 × 10^−1^	0.900	9.7 × 10^−2^	0.868
	35	1.5 × 10^−1^	0.908	3.2 × 10^−1^	0.899	1.9 × 10^−1^	0.678
	45	2.4 × 10^−1^	0.999	5.0 × 10^−1^	0.799	5.0 × 10^−1^	0.824
First-order	4	0.5 × 10^−2^	0.808	0.9 × 10^−2^	0.844	1.2 × 10^−2^	0.716
	25	0.2 × 10^−2^	0.922	1.6 × 10^−2^	0.928	2.8 × 10^−2^	0.873
	35	3.2 × 10^−2^	0.950	8.0 × 10^−2^	0.919	7.5 × 10^−2^	0.972
	45	4.7 × 10^−2^	0.991	1.1 × 10^−1^	0.818	1.1 × 10^−1^	0.859

**Table 5:** Predicted shelf life of IML stored at 4 °C, 25 °C, 35 °C, and 45 °C in Al bag with nitrogen, Al bag without nitrogen, and clear plastic bag.

Packaging	Shelf Life, Weeks	4 °C	25 °C	35 °C	45 °C
Al bag with nitrogen	Actual	>24	15	12	3
	Predicted	58	14	7	4
Al bag without nitrogen	Actual	>24	15	<3	<3
	Predicted	23	14	3	2
Clear plastic bag	Actual	21	9	<3	<3
	Predicted	19	8	3	2

## Data Availability

The original contributions presented in this study are included in the article/Supplementary Materials. Further inquiries can be directed to the corresponding author.

## Supplementary Materials

The following supporting information can be downloaded at https://www.mdpi.com/article/10.3390/foods15010040/s1, Table S1: Water activities (a_w_) of IML packed in Al bag with nitrogen during storage time at different temperatures (4, 25, 35 and 45 °C); Table S2: Water activities (aw) of IML packed in Al bag without nitrogen during storage time at different temperatures (4, 25, 35 and 45 °C); Table S3: Water activities (aw) of IML packed in clear plastic bag during storage time at different temperatures (4, 25, 35 and 45 °C); Table S4: Moisture contents of IML packed in Al bag with nitrogen during storage time at different temperatures (4, 25, 35 and 45 °C); Table S5: Moisture contents of IML packed in Al bag without nitrogen during storage time at different temperatures (4, 25, 35 and 45 °C); Table S6: Moisture contents of IML packed in clear plastic bag during storage time at different temperatures (4, 25, 35 and 45 °C); Table S7: ANOVA for water activities and moisture contents of intermediate moisture longan during storage; Table S8: ANOVA for color analysis of intermediate moisture longan during storage; Table S9: ANOVA for texture analysis of intermediate moisture longan during storage; Table S10: ANOVA for sensory evaluation scores of intermediate moisture longan during storage period.

## Abbreviations

The following abbreviations are used in this manuscript:

Al	Aluminum
a_w_	Water activity
BLGBB	Brilliant Green Lactose Bile Broth
IML	Intermediate Moisture Longan
MAP	Modified Atmosphere Packaging
MC	Moisture Content
OD	Osmotic Dehydration
PCA	Plate Count Agar
PDA	Potato Dextrose Agar
LSB	Lactose Broth
TBC	Total Bacterial Count
YMC	Yeast and Mold Count
