# Supplementary material for: Shelf Life Prediction of Longan with Intermediate Moisture Using Osmotic Dehydration, Combined with Different Packaging and Storage Temperatures
Source: Foods. 2025 Dec 23;15(1):40. doi: 10.3390/foods15010040 (PMC12785622; doi:10.3390/foods15010040)
Supplement: Supplementary file 1 [file foods-15-00040-s001.zip › foods-3960833-supplementary.pdf]

**Table S1** Water activities ($a_w$) of IML packed in Al bag with nitrogen during storage time at different temperatures (4, 25, 35 and 45 °C).

| Storage period (weeks) | Al bag with nitrogen | | | |
|---|---|---|---|---|
| | 4°C | 25°C | 35°C | 45°C |
| 0 | 0.600 ± 0.020 | 0.600 ± 0.020 | 0.600 ± 0.020 | 0.600 ± 0.020 |
| 2 | 0.604 ± 0.038 | 0.612 ± 0.007 | 0.653 ± 0.038 | 0.656 ± 0.004 |
| 4 | 0.601 ± 0.042 | 0.627 ± 0.008 | 0.648 ± 0.033 | 0.653 ± 0.036 |
| 6 | 0.614 ± 0.017 | 0.628 ± 0.018 | 0.622 ± 0.062 | 0.640 ± 0.020 |
| 8 | 0.616 ± 0.010 | 0.636 ± 0.030 | 0.631 ± 0.021 | 0.636 ± 0.019 |
| 10 | 0.619 ± 0.021 | 0.636 ± 0.005 | 0.633 ± 0.014 | 0.635 ± 0.022 |
| 12 | 0.621 ± 0.014 | 0.647 ± 0.010 | 0.628 ± 0.013 | 0.630 ± 0.008 |
| 14 | 0.622 ± 0.018 | 0.648 ± 0.031 | 0.630 ± 0.009 | 0.628 ± 0.014 |
| 16 | 0.622 ± 0.010 | 0.666 ± 0.041 | 0.630 ± 0.017 | 0.606 ± 0.043 |
| 18 | 0.626 ± 0.009 | 0.660 ± 0.038 | - | - |
| 20 | 0.626 ± 0.013 | 0.643 ± 0.017 | - | - |
| 22 | 0.626 ± 0.012 | 0.641 ± 0.005 | - | - |
| 24 | 0.620 ± 0.039 | 0.684 ± 0.029 | - | - |

- no measurement because shelf – life was ended.

**Table S2** Water activities (aw) of IML packed in Al bag without nitrogen during storage time at different temperatures (4, 25, 35 and 45 °C).

| Storage period (weeks) | Al bag without nitrogen | | | |
|---|---|---|---|---|
| | 4°C | 25°C | 35°C | 45°C |
| 0 | 0.600 ± 0.020 | 0.600 ± 0.020 | 0.600 ± 0.020 | 0.600 ± 0.020 |
| 2 | 0.663 ± 0.030 | 0.634 ± 0.024 | 0.625 ± 0.070 | 0.610 ± 0.009 |
| 4 | 0.665 ± 0.025 | 0.655 ± 0.035 | 0.629 ± 0.074 | 0.607 ± 0.039 |
| 6 | 0.668 ± 0.047 | 0.661 ± 0.038 | 0.630 ± 0.031 | 0.590 ± 0.055 |
| 8 | 0.684 ± 0.025 | 0.671 ± 0.006 | 0.627 ± 0.017 | 0.582 ± 0.034 |
| 10 | 0.689 ± 0.011 | 0.691 ± 0.015 | 0.622 ± 0.016 | 0.552 ± 0.007 |
| 12 | 0.692 ± 0.017 | 0.701 ± 0.012 | 0.619 ± 0.009 | 0.550 ± 0.009 |
| 14 | 0.689 ± 0.012 | 0.719 ± 0.040 | 0.583 ± 0.009 | 0.526 ± 0.006 |
| 16 | 0.710 ± 0.006 | 0.732 ± 0.055 | 0.582 ± 0.010 | 0.511 ± 0.019 |
| 18 | 0.714 ± 0.031 | 0.714 ± 0.006 | - | - |
| 20 | 0.720 ± 0.040 | 0.716 ± 0.008 | - | - |
| 22 | 0.728 ± 0.017 | 0.715 ± 0.015 | - | - |
| 24 | 0.732 ± 0.039 | 0.715 ± 0.004 | - | - |

- no measurement because shelf – life was ended.

**Table S3** Water activities (aw) of IML packed in clear plastic bag during storage time at different temperatures (4, 25, 35 and 45 °C).

| Storage period (weeks) | Clear plastic bag | | | |
|---|---|---|---|---|
| | 4°C | 25°C | 35°C | 45°C |
| 0 | 0.600 ± 0.020 | 0.600 ± 0.020 | 0.600 ± 0.020 | 0.600 ± 0.020 |
| 2 | 0.635 ± 0.023 | 0.656 ± 0.028 | 0.645 ± 0.021 | 0.617 ± 0.038 |
| 4 | 0.665 ± 0.037 | 0.661 ± 0.040 | 0.641 ± 0.017 | 0.579 ± 0.004 |
| 6 | 0.666 ± 0.009 | 0.674 ± 0.026 | 0.639 ± 0.020 | 0.573 ± 0.038 |
| 8 | 0.670 ± 0.008 | 0.681 ± 0.011 | 0.603 ± 0.008 | 0.570 ± 0.044 |
| 10 | 0.677 ± 0.009 | 0.697 ± 0.009 | 0.603 ± 0.038 | 0.526 ± 0.008 |
| 12 | 0.696 ± 0.011 | 0.703 ± 0.007 | 0.589 ± 0.004 | 0.523 ± 0.037 |
| 14 | 0.699 ± 0.012 | 0.709 ± 0.057 | 0.549 ± 0.018 | 0.409 ± 0.007 |
| 16 | 0.705 ± 0.036 | 0.724 ± 0.049 | 0.533 ± 0.004 | 0.360 ± 0.013 |
| 18 | 0.703 ± 0.062 | 0.718 ± 0.016 | - | - |
| 20 | 0.715 ± 0.005 | 0.717 ± 0.018 | - | - |
| 22 | 0.710 ± 0.069 | 0.720 ± 0.009 | - | - |
| 24 | 0.724 ± 0.033 | 0.726 ± 0.050 | - | - |

- no measurement because shelf – life was ended.

**Table S4** Moisture contents of IML packed in Al bag with nitrogen during storage time at different temperatures (4, 25, 35 and 45 °C).

| **Storage period (weeks)** | **Al bag with nitrogen** | | | |
|---|---|---|---|---|
| | **4°C** | **25°C** | **35°C** | **45°C** |
| 0 | 23.58 ± 1.68 | 23.58 ± 1.68 | 23.58 ± 1.68 | 23.58 ± 1.68 |
| 2 | 27.46 ± 3.78 | 23.04 ± 2.36 | 26.52 ± 1.60 | 32.77 ± 6.35 |
| 4 | 27.47 ± 3.36 | 25.06 ± 3.07 | 24.71 ± 6.40 | 28.15 ± 0.82 |
| 6 | 27.60 ± 3.93 | 25.76 ± 2.95 | 23.30 ± 0.52 | 27.74 ± 1.42 |
| 8 | 28.91 ± 3.05 | 27.06 ± 1.42 | 22.33 ± 2.22 | 24.52 ± 2.78 |
| 10 | 29.51 ± 2.03 | 32.37 ± 1.58 | 21.30 ± 0.95 | 23.26 ± 1.58 |
| 12 | 28.21 ± 1.11 | 32.82 ± 2.02 | 20.23 ± 4.10 | 22.60 ± 2.69 |
| 14 | 27.29 ± 0.84 | 34.63 ± 1.87 | 19.46 ± 5.67 | 22.00 ± 1.36 |
| 16 | 30.99 ± 1.27 | 36.54 ± 2.04 | 18.57 ± 2.59 | 19.81 ± 7.80 |
| 18 | 31.53 ± 4.10 | 37.16 ± 1.59 | - | - |
| 20 | 31.85 ± 2.47 | 37.28 ± 3.97 | - | - |
| 22 | 32.30 ± 0.53 | 38.78 ± 0.45 | - | - |
| 24 | 32.32 ± 1.39 | 39.05 ± 1.22 | - | - |

- no measurement because shelf – life was ended.

**Table S5** Moisture contents of IML packed in Al bag without nitrogen during storage time at different temperatures (4, 25, 35 and 45 °C).

| Storage period (weeks) | Al bag without nitrogen | | | |
|---|---|---|---|---|
| | 4°C | 25°C | 35°C | 45°C |
| 0 | 23.58 ± 1.68 | 23.58 ± 1.68 | 23.58 ± 1.68 | 23.58 ± 1.68 |
| 2 | 27.08 ± 6.44 | 23.15 ± 4.08 | 24.63 ± 3.43 | 24.25 ± 1.79 |
| 4 | 27.66 ± 2.49 | 25.15 ± 4.47 | 23.61 ± 5.57 | 23.15 ± 1.83 |
| 6 | 28.71 ± 4.73 | 25.26 ± 1.63 | 23.46 ± 0.54 | 19.72 ± 4.10 |
| 8 | 29.98 ± 8.47 | 26.55 ± 4.20 | 22.45 ± 1.51 | 19.90 ± 0.53 |
| 10 | 30.57 ± 1.41 | 29.12 ± 1.05 | 22.40 ± 0.93 | 19.89 ± 0.53 |
| 12 | 31.10 ± 0.86 | 29.91 ± 1.55 | 21.64 ± 0.71 | 18.60 ± 3.46 |
| 14 | 31.43 ± 1.43 | 33.03 ± 1.42 | 20.32 ± 1.57 | 18.32 ± 6.95 |
| 16 | 31.74 ± 1.40 | 34.54 ± 1.60 | 19.98 ± 4.50 | 18.10 ± 0.84 |
| 18 | 32.46 ± 2.70 | 34.56 ± 2.24 | - | - |
| 20 | 32.58 ± 0.54 | 38.83 ± 1.39 | - | - |
| 22 | 32.93 ± 0.82 | 39.51 ± 1.48 | - | - |
| 24 | 33.25 ± 1.56 | 40.35 ± 4.13 | - | - |

- no measurement because shelf – life was ended.

**Table S6** Moisture contents of IML packed in clear plastic bag during storage time at different temperatures (4, 25, 35 and 45 °C).

| Storage period (weeks) | Clear plastic bag | | | |
|---|---|---|---|---|
| | 4°C | 25°C | 35°C | 45°C |
| 0 | 23.58 ± 1.68 | 23.58 ± 1.68 | 23.58 ± 1.68 | 23.58 ± 1.68 |
| 2 | 25.88 ± 3.03 | 24.37 ± 1.59 | 25.84 ± 3.28 | 22.93 ± 2.02 |
| 4 | 26.67 ± 2.11 | 24.36 ± 1.68 | 23.15 ± 2.33 | 21.33 ± 1.55 |
| 6 | 26.38 ± 3.73 | 25.78 ± 2.67 | 21.17 ± 2.83 | 19.40 ± 2.00 |
| 8 | 26.28 ± 0.86 | 27.10 ± 0.82 | 20.40 ± 1.35 | 19.23 ± 1.05 |
| 10 | 26.62 ± 1.19 | 29.18 ± 1.10 | 17.80 ± 3.35 | 15.51 ± 0.40 |
| 12 | 28.01 ± 3.06 | 29.22 ± 2.63 | 17.42 ± 0.82 | 14.01 ± 0.38 |
| 14 | 29.16 ± 1.57 | 31.25 ± 0.80 | 17.24 ± 1.33 | 9.04 ± 0.31 |
| 16 | 29.91 ± 0.32 | 31.93 ± 2.14 | 15.97 ± 0.45 | 8.75 ± 0.87 |
| 18 | 30.31 ± 2.88 | 33.76 ± 2.45 | - | - |
| 20 | 31.32 ± 0.92 | 35.44 ± 4.26 | - | - |
| 22 | 31.98 ± 1.54 | 40.95 ± 1.02 | - | - |
| 24 | 32.14 ± 2.09 | 44.07 ± 13.5 | - | - |

- no measurement because shelf – life was ended.

**Table S7** ANOVA for water activities and moisture contents of intermediate moisture longan during storage.

| Source of variation | df | Mean square | | | |
|---|---|---|---|---|---|
| | | Water activity | P - value | Moisture content | P - value |
| Temperature of storage (TS) | 3 | 0.200 | 0.000 | 2012.11 | 0.000 |
| Packaging types (PT) | 2 | 0.011 | 0.002 | 416.68 | 0.000 |
| Storage period (SP) | 12 | 0.014 | 0.000 | 356.54 | 0.000 |
| Effect of (TS x PT) | 6 | 0.079 | 0.000 | 149.54 | 0.000 |
| Error | | 0.002 | | 17.44 | |

**Table S8** ANOVA for color analysis of intermediate moisture longan during storage.

| Source of variation | df | Mean square | | | | | |
|---|---|---|---|---|---|---|---|
| | | L* | P - value | a* | P - value | b* | P - value |
| Temperature of storage (TS) | 3 | 3150.70 | 0.000 | 14.65 | 0.000 | 1255.84 | 0.000 |
| Packaging types (PT) | 2 | 1647.65 | 0.000 | 23.45 | 0.000 | 137.37 | 0.000 |
| Storage period (SP) | 12 | 842.88 | 0.000 | 101.71 | 0.000 | 291.11 | 0.000 |
| Effect of (TS x PT) | 6 | 550.72 | 0.000 | 6.37 | 0.000 | 42.47 | 0.000 |
| Error | | 4.60 | | 1.53 | | 3.01 | |

**Table S9** ANOVA for texture analysis of intermediate moisture longan during storage.

| Source of variation | df | Mean square | |
|---|---|---|---|
| | | Hardness | P - value |
| Temperature of storage (TS) | 3 | 110.97 | 0.000 |
| Packaging types (PT) | 2 | 6.841 | 0.000 |
| Storage period (SP) | 12 | 36.44 | 0.000 |
| Effect of (TS x PT) | 6 | 2.12 | 0.004 |
| Error | | 0.65 | |

**Table S10** ANOVA for sensory evaluation scores of intermediate moisture longan during storage period.

| Source of variation | df | Mean square | | | | | |
|---|---|---|---|---|---|---|---|
| | | Overall acceptance | P – value | Color | P - value | Odor | P - value |
| Temperature of storage (TS) | 3 | 811.93 | 0.000 | 1186.74 | 0.000 | 511.53 | 0.000 |
| Packaging types (PT) | 2 | 124.35 | 0.000 | 210.35 | 0.000 | 51.13 | 0.000 |
| Storage period (SP) | 8 | 97.82 | 0.000 | 78.50 | 0.000 | 78.39 | 0.000 |
| Effect of (TS x PT) | 6 | 6.62 | 0.012 | 22.33 | 0.000 | 6.34 | 0.023 |
| Error | | 2.44 | | 2.43 | | 2.59 | |
